# Unveiling the immune microenvironment of complex tissues and tumors in transcriptomics through a deconvolution approach

**DOI:** 10.1186/s12885-025-14089-w

**Published:** 2025-05-01

**Authors:** Shu-Hwa Chen, Bo-Yi Yu, Wen-Yu Kuo, Ya-Bo Lin, Sheng-Yao Su, Wei-Hsuan Chuang, I.-Hsuan Lu, Chung-Yen Lin

**Affiliations:** 1 TMU Research Center of Cancer Translational Medicine, Taipei Medical University, 250 Wu-Xing Street, Taipei, Taiwan; 2 Research Center for Advanced Science and Technology, the University of Tokyo, 4-6-1 Komaba, Meguro-Ku, Tokyo, 153-8904 Japan; 3 Institute of Information Science, Academia Sinica, 128 Academia Road, Section 2, Nankang, Taipei 115 Taiwan; 4 Department of Smart Computing and Applied Mathematics, Tunghai University, Taichung City, 407224 Taiwan; 5 Institute of Fisheries Science, National Taiwan University, Taipei, Taiwan; 6 Genome and Systems Biology Degree Program, National Taiwan University, Taipei, Taiwan

**Keywords:** Cancer, Immunotherapy, Deconvolution, Alpha diversity, Beta diversity, Precision medicine, Immune microenvironment

## Abstract

Accurately resolving the composition of tumor-infiltrating leukocytes is pivotal for advancing cancer immunotherapy strategies. Despite the success of some clinical trials, applying these strategies remains limited due to the challenges in deciphering the immune microenvironment. In this study, we developed a streamlined, two-step workflow to address the complexity of bioinformatics processes involved in analyzing immune cell composition from transcriptomics data. Our dockerized toolkit, DOCexpress_fastqc, integrates the hisat2-stringtie pipeline with customized scripts within Galaxy/Docker environments, facilitating RNA sequencing (RNA-seq) gene expression profiling. The output from DOCexpress_fastqc is seamlessly formatted with mySORT, a web application that employs a deconvolution algorithm to determine the immune content across 21 cell subclasses. We validated mySORT using synthetic pseudo-bulk data derived from single-cell RNA sequencing (scRNA-seq) datasets. Our predictions exhibit strong concordance with the ground-truth immune cell composition, achieving Pearson’s correlation coefficients of 0.871 in melanoma patients and 0.775 in head and neck cancer patients. Additionally, mySORT outperforms existing methods like CIBERSORT in accuracy and provides a wide range of data visualization features, such as hierarchical clustering and cell complexity plots. The toolkit and web application are freely available for the research community, providing enhanced resolution for conventional bulk RNA sequencing data and facilitating the analysis of immune microenvironment responses in immunotherapy. The mySORT demo website and Docker image are free at https://mysort.iis.sinica.edu.tw and https://hub.docker.com/r/lsbnb/mysort_2022.

**Supplementary Information:**

The online version contains supplementary material available at 10.1186/s12885-025-14089-w.

## Introduction

Cancer is a disease involving the regulation of cell proliferation. It is characterized by an unanticipated cell population expansion, which consumes body space and resources and may acquire the ability to recolonize (i.e., metastasize) to distant sites. Although the immune system can eliminate foreign pathogens and abnormal cells, cancer cells often manage to evade detection. Recent discoveries regarding the immune blockage/checkpoint programmed cell death protein 1 and cytotoxic T-lymphocyte-associated protein 4 pathways [1] have led to a novel cancer therapy paradigm [2–4]. Other studies have also reported adverse side effects and indicated that responses to treatments with checkpoint inhibitors vary and that not all cancer types or cases are compatible with these two pathways [5, 6]. These diverse responses of patients and cancer types may be attributable to the composition of immune cells [7–9]. Laboratory techniques such as flow cytometry and immunocytochemistry may provide a resolution based on a limited number of known biomarkers. However, developing a scalable, robust approach for resolving all immune cell types remains difficult. High-throughput technologies such as microarrays and next-generation sequencing (NGS) have revolutionized the field of gene expression profiling, enabling the development of methods for estimating the composition of immune cells based on transcript profiling [10–17]. Multiple methods, such as quadratic programming [11], the digital sorting algorithm [12], and semisupervised nonnegative matrix factorization [13], have been proposed for resolving cellular components from microarray data. Newman et al. proposed a novel strategy [14] for selecting gene features. They implemented their method to a web service, CIBERSORT, by comparing the cellular composition result with the ground truth (e.g., cell fractions, flow cytometry typing/ classification) [18]. Nevertheless, accurately estimating specific immune cell types remains a significant unresolved challenge, as demonstrated by the root mean square errors and correlation coefficients presented in their study. In this study, we revisited these datasets, excluded cell-type subgroups with low consistency, optimized the signature gene set, and proposed an algorithm called mySORT. This method addressed the poor performance of a specific cell type [19] and was used with RNA sequencing (RNA-seq) data [20]. To date, an increasing number of tools have been developed for determining the composition of immune cells [21].

Single-cell RNA sequencing combines cell isolation techniques with advancements in microfluidics and sequencing technologies. By providing transcriptome profiling at the single-cell level, it effectively uncovers the complexity and heterogeneity of cellular composition, establishing itself as a powerful tool for investigating the tumor immune microenvironment [22–26]. However, tissue dissociation can be challenging for solid tumors, and the high costs and time-intensive processes further limit its feasibility for clinical applications. Importantly, methods resolving the composition of immune cells from bulk transcriptomics, such as mySORT, remain accessible and reliable strategies for understanding the tumor microenvironment.

For most bench scientists, dealing with the entire bioinformatic processes of immune-cell composition necessitates diverse knowledge, including selecting hardware, software, and cloud-based computational resources and troubleshooting skills. Pipelines may take on different forms, such as a command line scripting toolkit, a Galaxy workflow, or a dockerized image. Users can find online tutorials and manage RNA-seq data from raw reads. By contrast, other factors, such as file format incompatibility or conflicts in the genome reference version and gene annotation used in each step, may complicate the movement of data analysis processes. Therefore, intuitive, flexible, and scalable pipelines (predefined or user-defined) that can complete entire tasks are required.

This study developed a two-step workflow, comprising DOCexpress_fastqc and mySORT web, providing an integrated solution to address challenges in large-scale data processing, cross-program compatibility, and reference version control. DOCexpress_fastqc is a Dockerized RNA-seq processing toolkit built on Galaxy's open architecture with a user-friendly interface (UI) and enhanced user experience (UX). Its outputs are specifically formatted to match the input requirements of mySORT web, ensuring seamless data transfer between tools and minimizing issues caused by incompatible file formats. MySORT web application computes the relative proportions of twenty-one immune cell types from the input data. It visualizes immune cells diversity metrics, including clustering, alpha-diversity, and beta-diversity analyses, through various plots. These plots highlight the complexity of immune cell populations, providing valuable insights into variability within and across samples. This two-step pipeline facilitates the immune cell deconvolution analysis, promoting consistency, reproducibility, and efficient data processing. To demonstrate the utility and reliability of this approach, we applied it to single-cell RNA-seq data, validating the performance and consistency of the mySORT web analysis.

## Materials and methods

### Estimation of RNA-Seq data in a galaxy web interface from a docker image

To simplify the process of RNA-seq analysis from raw reads to expression profiling, we implemented a pipeline based on hisat2-stringtie [27] and Ballgown [28] with a Galaxy/Docker image (https://github.com/bgruening/docker-galaxy-stable). Generally, the workflow in expression profiling estimation involves the determination of (1) fragments per kilobase per million (FPKM) in transcripts, (2) FPKM in gene symbols, and (3) transcripts per million (TPM) in gene symbols with pair- or single-end sequencing. Our pipeline included fastqc (https://github.com/s-andrews/FastQC) to provide a quality check for each dataset in a graphical view through Galaxy. Finally, several scripts developed by our team were used to merge the transcriptome of each sample into a single table in a Comma-Separated Values (CSV) file with Ensembl transcripts or gene symbols are the primary key.

### Construction of synthetic pseudo-bulk gene expression data

The single-cell RNA-seq data of melanoma and head and neck cancer patients were downloaded from the NCBI GEO dataset (accession numbers GSE72056 and GSE103322, respectively) [24, 25]. Both single-cell datasets were generated using the Smart-seq2 protocol. Preprocessed gene expression profiles containing TPM values were retrieved from the GEO database without additional processing steps. Due to the variability across single-cell datasets, we adhered to each study's data quality control recommendations. Specifically, high-quality cells were maintained with at least 1,700 expressed genes for melanoma samples and 2,000 expressed genes for head and neck squamous cell carcinoma (HNSCC) samples. Adequate housekeeping gene expression was also confirmed in every qualified cell. Finally, 4,640 cells from the melanoma dataset and 5,901 cells from the HNSCC dataset passed the quality control. Pseudo-bulk gene expression profiles were then constructed by averaging the gene expression profiles of each patient.

### Calculation of the ground truth of immune-cell composition

Only four immune-cell types were initially identified in the melanoma dataset: B cells, T cells, natural killer cells, and macrophages. Each cell's CD4, CD8A, and CD8B gene expression divided the T-cell cluster into CD4 and CD8 T cells. T cells with *CD4* expression and no *CD8A* or *CD8B* expression were assigned to the CD4 T-cell cluster, whereas T cells with *CD8A* and *CD8B* expression and no *CD4* expression were classified as CD8 T cells. Because of the extremely low immune cells, two melanoma samples were discarded, leaving 17 samples for downstream analysis. Finally, the ground truth of the relative immune-cell proportion was calculated based on the authors' cell identity in each sample. Similarly, the strategy described earlier was applied to the HNSCC dataset. Six immune-cell types were used: B cells, CD4 T cells, CD8 T cells, macrophages, dendritic cells, mast cells, and 16 qualified HNSCC samples.

### Comparison of the estimated and true immune-cell composition

Because the single-cell data and the output of mySORT shared several immune-cell types, the sum of the ground-truth value and predicted value was rescaled to 1 as the total value for comparison. Pearson's correlation coefficient and the root-mean-square value were then used to determine the correlation and difference between the estimated and actual immune-cell content.

### Validation by the scRNA-seq data of NSCLC patients

The single-cell RNA sequencing data of NSCLC patients was downloaded from NCBI GEO Accession GSE148071 [29]. Following the instructions of the original publication, we removed low-quality cells by these four rules: (1) lower than 200 expressed genes, (2) higher than 5000 expressed genes, (3) more than 30,000 UMIs, and (4) mitochondria content higher than 30%. Among all 42 NSCLC patients, we used patients with higher than 20% immune cell content for better validation. Finally, 34,895 cells from 23 patients were applied to construct the pseudo-bulk gene expression profiles. The relative immune cell proportion of four cell types (T, B, mast, and neutrophils) was calculated based on the original article's cell clustering and classification results. To compare the ground truth and predicted immune cell content of mySORT, we scaled the sum of both the ground-truth value and the predicted value to 1 as the total value. The Pearson correlation coefficient and root-mean-square were then used to measure the correlation and deviations between the estimation of immune cell content and the ground truth.

### System implementation

To improve the user experience, mySORT was constructed using a Linux-Apach-MySQL-PHP (LAMP) system architecture, which is comprised of Linux Ubuntu 16.04, Apache 2.04, MySQL 5.7, PHP 5.1 with a Bootstrap 3 Crown Commercial Service (CSS) framework (http://getbootstrap.com/), jQuery 1.11.1, and jQuery Validation v1.17. The core of the analytical process was implemented in R software (version 3.4.2). Subsequently, mySORT was run in a virtual machine (16 core vCPU at 2.27-GHz, 64 GB of RAM, and 500 GB of storage) on the Institute of Information Science, Academia Sinica (Taipei, Taiwan) cloud infrastructure.

## Results and discussions

### Estimating and merging the expression profiles for mySORT

A graphical web platform was implemented to guide the expression profiling process wherein the hisat2-stringtie pipeline and our scripts were integrated on Galaxy/Docker to facilitate dealing with large amounts of NGS data. The users were required to prepare the raw reads (paired or single ends) generated from RNA-seq and download the genome sequence in a general feature format for humans and mice. This application, called DOCexpress_fastqc (https://hub.docker.com/r/lsbnb/docexpress_fastqc), can merge all the transcriptomes in FPKM or TPM based on gene symbols or transcripts as input for mySORT and is available on Docker Hub. DOCexpress_fastqc is an open-source, Galaxy-based, intuitive, scalable, and comprehensive platform available for the biomedical research community. Details regarding the deployment and use of this platform are available at https://hub.docker.com/r/lsbnb/docexpress_fastqc.

**Fig. 1:**
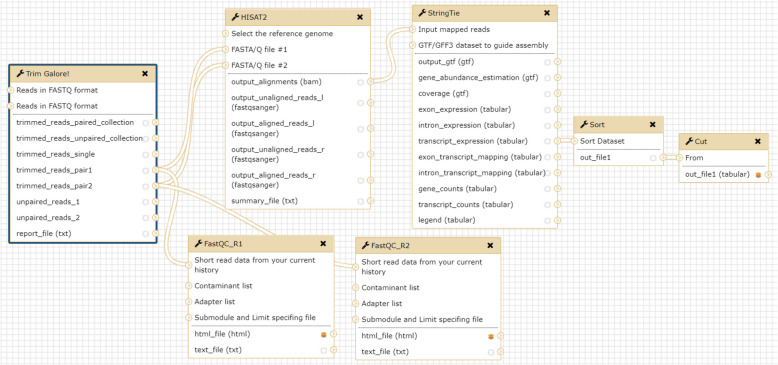
Pipeline of expression profiling estimation sorted by the gene symbol for RNA-Seq in paired ends

**Fig. 2:**
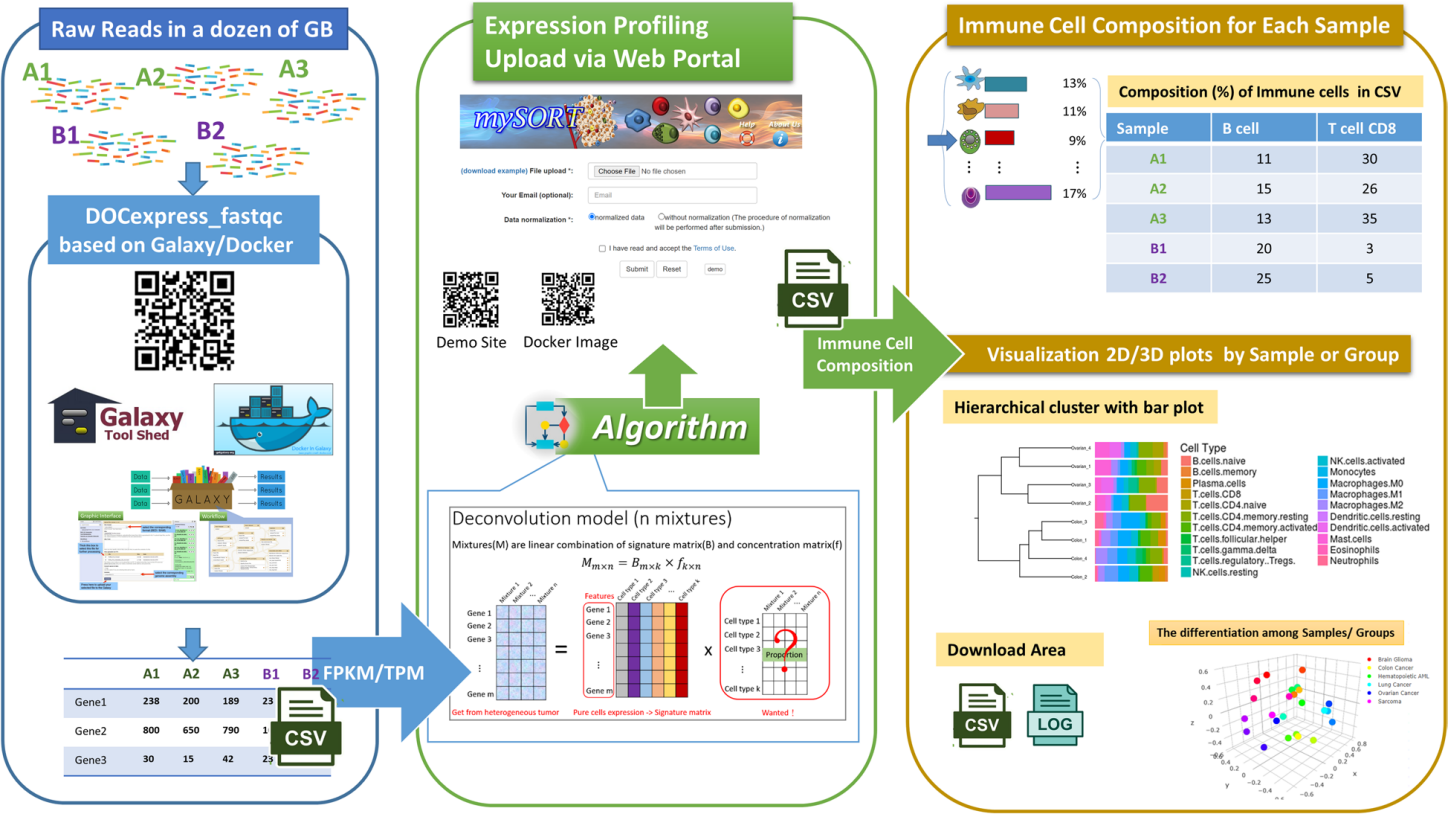
Workflow from raw reads to immune-cell composition

The output of DOCexpress_fastqc can be used by many tools on locally installed or cloud versions of Galaxy/Docker to perform deep analyses of differential expression (Fig. 1). If the users add Ballgown or DESeq2/edgeR/limma-voom [30] to Galaxy/Docker with DOCexpress_fastqc, they can estimate differential expression by gene symbols and Ensemble transcripts through the web interface (the block of Fig. 2 in blue). DOCexpress_fastqc can also run on a local machine or cloud and can be integrated into a platform load-sharing facility or Portable Batch System (PBS) professional (OpenPBS, http://www.pbspro.org/) clusters.

### Usage of mySORT Web

Generally, users can upload a text or CSV file containing a gene expression matrix of single or multiple samples generated from DocExpress_fastqc. The matrix should contain gene symbols as rows and sample names as columns. If the submitted expressed data have not been previously normalized (user-defined), then mySORT performs a log transformation. For more information regarding the algorithm of mySORT with pseudocodes, please refer to our previous publication [19]. To simplify the operation of mySORT, a demo was prepared, which included several expression data from different types of cancer tissues (the block of Fig. 2 in green). The users categorized these expression datasets into several groups for deep analysis. Simultaneously, the users visualized alpha and beta diversity on low-dimensional 2D or 3D plots. Calculation and visualization were performed using the R packages *Vegan* [31], *Phyloseq* [32], and *Plotly* (https://plot.ly).

As shown in the golden block of Fig. 2, the output of mySORT included four parts:

1. Immune Cell composition for each sample: A proportional table and a stacked bar chart of 21 immune-cell types
2. Visualization with bar plot: Hierarchical clustering of samples based on the immune-cell composition
3. The differentiation among samples: Visualization plots based on alpha and beta diversity
4. Download Area: Several text files in the download area for the original submission, results, alpha and beta diversity, signature matrix, and log file (please refer to the results page on the website)

As shown in Fig. 2, DOCexpress_fastqc guided the expression profiling process by integrating the hisat2-stringtie pipeline and our scripts on Galaxy/Docker from raw reads at the Giga Byte size level. The mySORT web-based tool was then used to perform a deconvolution analysis of the input expression profile generated by DOCexpress_fastqc. After completing the analytical process, mySORT provided a comparative immune-cell composition table and several visualized figures of hierarchical clustering, alpha-diversity, and beta-diversity analyses with several output files in the download area.

### Visualization plots of alpha and beta diversity

Alpha- and beta-diversity are used in comparing population components diversity within and between samples [33, 34]. The tumor immune microenvironment, characterized by its complexity and diversity in cell types, can be examined using these metrics. Here, Simpson's alpha-diversity index was adopted in our study to indicate the sample heterogeneity and the richness and evenness of immune-cell species. Alpha diversity measures the diversity within a single sample, assessing the number of different immune cell types (richness) and their proportions (evenness). It provides insight into how diverse and balanced a tissue's immune cell populations are within a tissue. Low alpha diversity would imply a simpler composition and/or dominance by fewer cell types. Additionally, a nonmetric multidimensional scaling (NMDS) plot was used to describe beta diversity, representing variations in immune cell composition between samples. Low beta diversity suggests a high similarity between samples in both immune cell composition and relative proportions.

Table 1 presents the characteristics of the web applications of mySORT and CIBERSORT for comparison. Although both web applications are easy to use, mySORT offers a more intuitive and comprehensive data visualization that allows for a glance at complex data and flexible options for deep analyses. In addition, regarding the RNA-seq deconvolution performance, mySORT is compatible with or even outperforms the currently available state-of-the-art deconvolution methods. This new version expands the mySORT deconvolution model to analyze single-cell RNA-seq data using blood biopsies, as described in the following section.

**Table 1:** Web Interface Functions and Result layouts of mySORT and CIBERSORT for Comparison

Functions and Outputs	mySORT	CIBERSORT
***User interface***		
Registration	**Not required**	Required
Custom signature matrix	Not allowed	**Allowed**
***Immune-cell composition***		
Relative proportion in the table	Yes	Yes
Stacked bar chart	Yes	Yes
Multiple data columns	Yes	Yes
***Data comparison***		
Hierarchical clustering	**Yes**	No
Cell diversity within a sample (alpha-diversity plot)	**Yes**	No
Cell diversity among samples (beta-diversity plot)	**Yes**	No
Data export	Yes	Yes

### Validation of mySORT performance by actual single-cell datasets

**Fig. 3:**
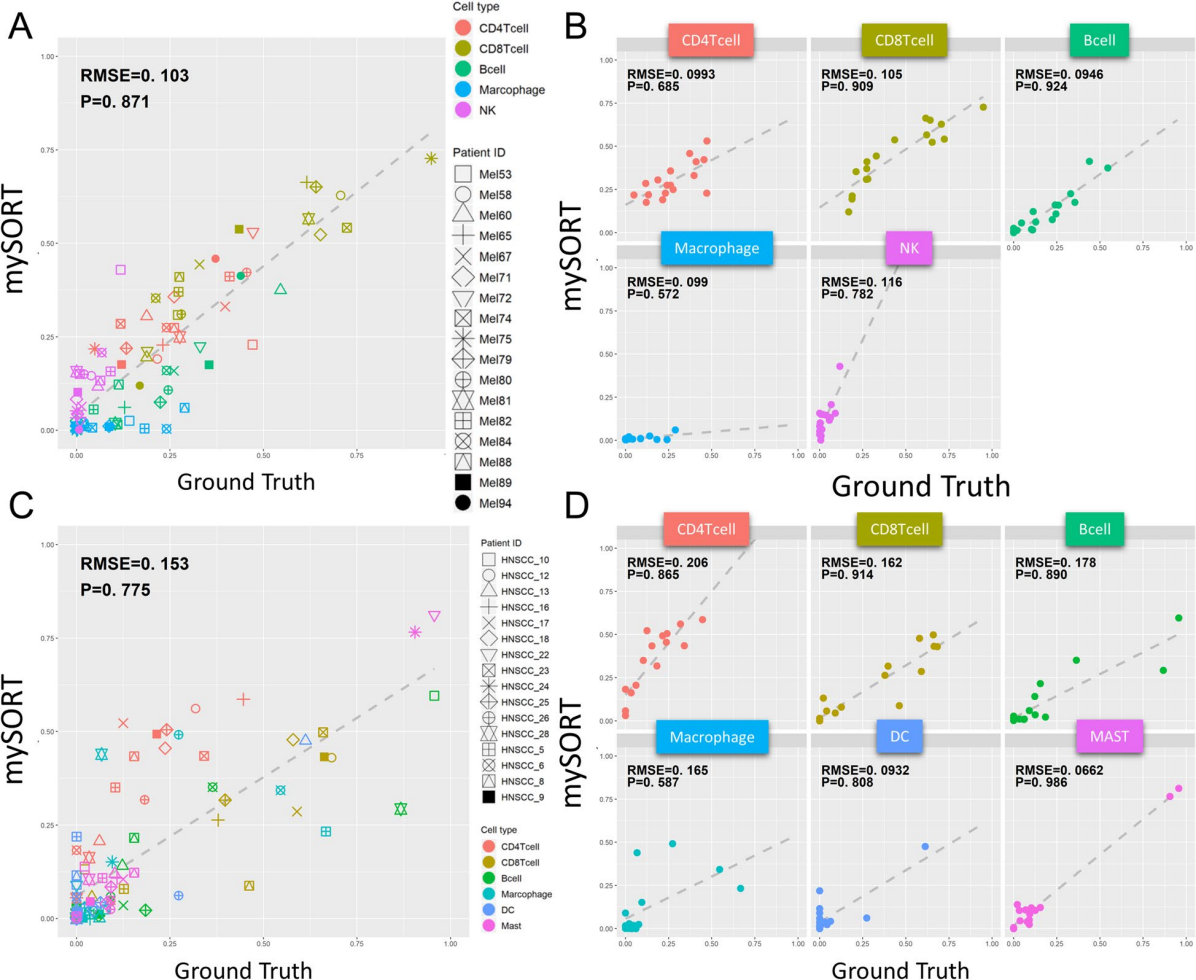
Correlation of the predicted values from mySORT and the ground truth. **A** Scatter plot of the predicted proportion of mySORT and ground truth from the melanoma dataset. The *x*-axis indicates the ground-truth values, and the *y*-axis indicates the predicted values of mySORT. The shapes represent the different patients, and the colors represent the different immune-cell types. **B** Scatter plot like that in (**A**) but based on each cell type only. **C**, **D** Scatter plots of the HNSCC dataset

Blood biopsies were used to benchmark the performance of mySORT among 20 adults, in whom nine immune-cell types were identified using flow cytometry. Our previous results indicated that the computational performance of mySORT was higher than that of CIBERSORT, a state-of-the-art deconvolution method. This superiority was evidenced by lower root mean square error values and higher Pearson’s correlation coefficients for most immune cell types. To further validate the performance of mySORT, a single-cell RNA-seq cutting-edge technology was used. Two public single-cell RNA-seq datasets of tumor samples were collected from 17 patients with melanoma and 16 patients with head and neck cancer. The synthetic pseudo-bulk data of the tumor samples were then used to estimate the relative proportion of immune-cell types. The results indicated that the prediction of mySORT strongly correlated with the ground truth in both datasets when all immune-cell types were considered (Fig. 3A and C). When the outcomes were separated by cell type, a strong correlation was observed in almost all cell types except for macrophages (Fig. 3B and D). This lower accuracy for macrophages may have been due to the low proportion of macrophage-based datasets in our signature matrix. Nevertheless, the overall performance of mySORT remained consistent with the single-cell data and was not considerably affected by this phenomenon. Integrating additional high-quality immune cell expression profiles from bulk or single-cell datasets is vital for enhancing the performance of our future model, particularly for predicting rare immune cell populations like macrophages. In the context of using massive and informative single-cell datasets, deep neural networks have the potential to further improve model performance due to the ability to capture complex relationships within high-dimensional data.

### Validation by the scRNA-seq data of NSCLC patients in mySORT

We tested the performance of mySORT by using another single-cell RNA-seq dataset of NSCLC patients [29]. We confirmed that our tool could also be applied to other scRNA-seq experiments conducted by a different platform and library preparation protocol. Cells of NSCLC patients were classified into various cell types, including six immune-cell types: T cells, B cells, mast cells, neutrophils, myeloid cells, and Follicular Dendritic Cells (fDCs) in the original article. To avoid the ambiguity of the definition of myeloid cells, we excluded them from the analysis. Also, fDCs were not used due to their rarity in tissues. Although the overall correlation coefficient was slightly lower than the previous two validation datasets, the individual immune-cell content of four immune cells (T cells, B cells, mast cells, neutrophils) still demonstrated the good performance of mySORT (0.632 – 0.902 correlation coefficients, Supplementary Fig. 1).

## Conclusion

Thanks to the advancements made in immunotherapy, novel anticancer drugs and therapies have been developed. Novel strategies for overcoming the immune suppressive capacity of cancer cells are expected to be developed in the future. The correlation between the cellular composition of cancer and drug response suggests that the heterogeneity of immune-cell populations is a critical problem in clinical practice. Therefore, we implemented mySORT into a user-friendly web framework in this study. We added two cell population diversity measurements to help biomedical researchers understand the tumor microenvironment of their samples by using comprehensive plots and charts.

While mySORT performs superior accuracy in the demonstrated microarray experiments compared to the state-of-the-art method CIBERSORT, its predictive power for certain cell types such as macrophages remained suboptimal. This limitation highlights the need for further refinement, particularly in handling rare immune cell populations. With the rapid development of single-cell sequencing technologies as well as knowledge of immunology, an increasing number of high-quality datasets have become publicly available. Our future work should focus on integrating these large-scale datasets into mySORT model training. Additionally, recent promising deep neural network-based architectures should be explored to the complexities of cell component deconvolution, further improving model performance. By combining these innovative approaches with the proposed model of mySORT, we may open new avenues for precision medicine applications in cancer immunotherapy.

## Availability and requirements

Project names: (1) DOCexpress_fastqc and (2) mySORT.

Project home pages:

(1) DOCexpress_fastqc: https://hub.docker.com/r/lsbnb/docexpress_fastqc. (2) Demo website for mySORT: http://mysort.iis.sinica.edu.tw/. Docker image: https://hub.docker.com/r/lsbnb/mysort_2022 (for academic use).

## Supplementary Information

Supplementary Material 1.

## Data Availability

This study used both the melanoma (NCBI GSE72056) and the head and neck cancer (NCBI GSE103322) datasets.

## Abbreviations

HNSCC: Head and neck squamous cell carcinoma
NMDS: Nonmetric multidimensional scaling
NGS: Next-generation sequencing
RNA-seq: RNA sequencing
FPKM: Fragments per kilobase per million
TPM: Transcripts per million
