# Supplementary material for: Unveiling the immune microenvironment of complex tissues and tumors in transcriptomics through a deconvolution approach
Source: BMC Cancer. 2025 May 1;25(Suppl 1):733. doi: 10.1186/s12885-025-14089-w (PMC12044707; doi:10.1186/s12885-025-14089-w)
Supplement: Supplementary file 1 — Supplementary Material 1. [file 12885_2025_14089_MOESM1_ESM.pdf]

A

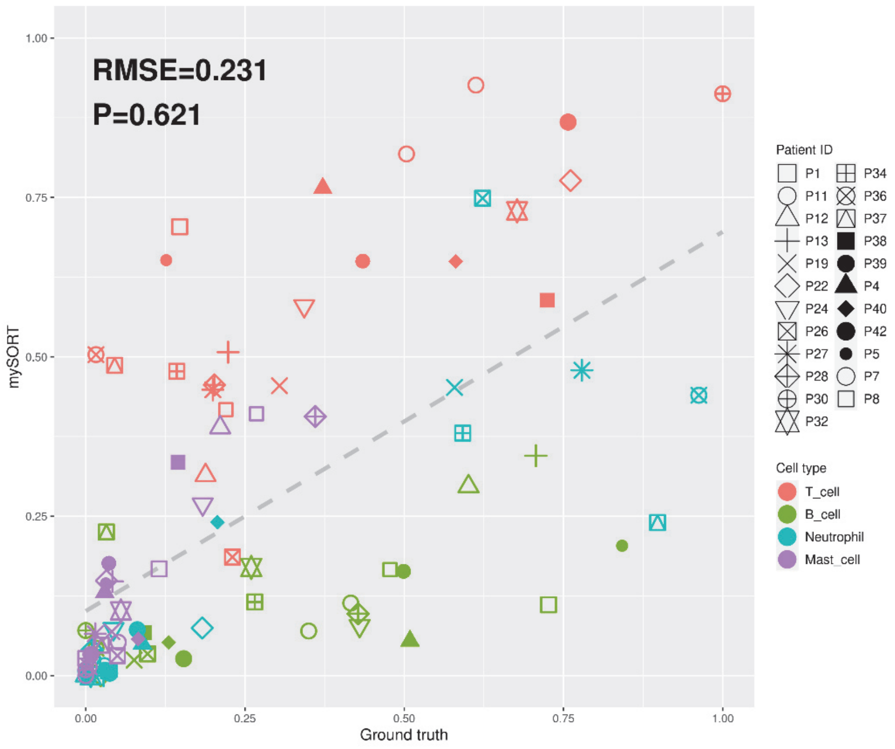
RMSE=0.231
P=0.621
mySORT
Ground truth
1.00
0.75
0.50
0.25
0.00
0.00
0.25
0.50
0.75
1.00
Patient ID
P1
P11
P12
P13
P19
P22
P24
P26
P27
P28
P30
P32
P34
P36
P37
P38
P39
P4
P40
P42
P5
P7
P8
Cell type
T_cell
B_cell
Neutrophil
Mast_cell

B

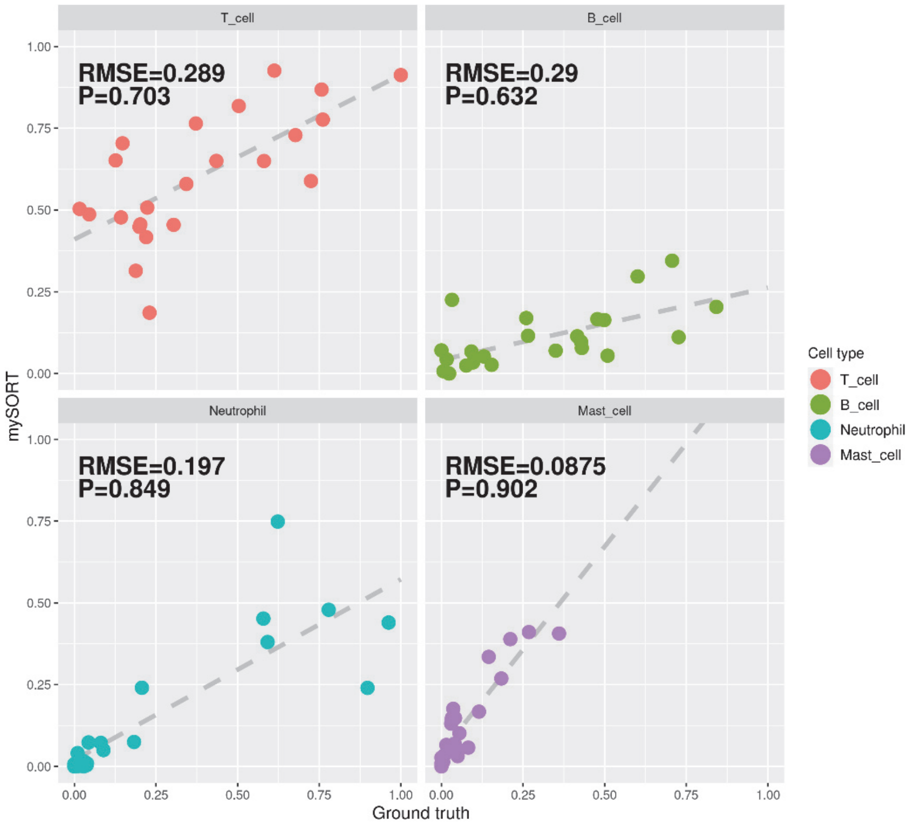
T_cell
B_cell
Neutrophil
Mast_cell
RMSE=0.289
P=0.703
RMSE=0.29
P=0.632
RMSE=0.197
P=0.849
RMSE=0.0875
P=0.902
mySORT
Ground truth
1.00
0.75
0.50
0.25
0.00
0.00
0.25
0.50
0.75
1.00
Cell type
T_cell
B_cell
Neutrophil
Mast_cell

Supplementary Figure 1. Correlation of the mySORT predicted values and the values in NSCLC patients.

(A) The scatter plot of the predicted immune-cell proportion of mySORT and the values from the non-small cell lung cancer dataset (GSE148071). The x-axis indicates the ground-truth value, and the y-axis indicates the predicted value of mySORT. Shapes represent the different patients, and colors represent the different immune cell types. (B) A similar scatter plot as (A) but plotted based on each cell type only.
